# EGFR-specific T cell frequencies correlate with EGFR expression in head and neck squamous cell carcinoma

**DOI:** 10.1186/1479-5876-9-168

**Published:** 2011-10-04

**Authors:** Patrick J Schuler, Petra Boeckers, Rainer Engers, Edwin Boelke, Murat Bas, Jens Greve, Claudia A Dumitru, Goetz F Lehnerdt, Robert L Ferris, Pedro A Andrade Filho, Sven Brandau, Stephan Lang, Theresa L Whiteside, Thomas K Hoffmann

**Affiliations:** 1 Universität Duisburg-Essen, Hals-Nasen-Ohrenklinik Essen, Germany; 2 Heinrich-Heine-Universität, Klinik für Chirurgie, Düsseldorf, Germany; 3 Heinrich-Heine-Universität, Institut für Pathologie, Düsseldorf, Germany; 4 Heinrich-Heine-Universität, Klinik für Strahlentherapie und Radioonkologie, Düsseldorf, Germany; 5 Technische Universität München, Hals-Nasen-Ohrenklinik, Klinikum Rechts der Isar, München, Germany; 6 University of Pittsburgh, Hillman Cancer Center, Pittsburgh, USA

**Keywords:** EGFR, head and neck carcinoma, tetramer, EGFR-specific T cells

## Abstract

**Background:**

In head and neck squamous cell carcinoma (HNSCC), expression levels of the epidermal growth factor receptor (EGFR) correlate with poor prognosis and decreased survival rates. As the mechanisms responsible for cellular immune response to EGFR *in vivo *remain unclear, the frequency and function of EGFR-specific cytotoxic T cells (CTL) was determined in HNSCC patients.

**Methods:**

The frequency of CTL specific for the HLA-A2.1-restricted EGFR-derived YLN peptide (YLNTVQPTCV) and KLF peptide (KLFGTSGQKT) was determined in 16 HLA-A2.1^+ ^HNSCC patients and 16 healthy HLA-A2.1^+ ^individuals (NC) by multicolor flow cytometry. Patients' results were correlated to EGFR expression obtained by immunohistochemistry in corresponding tumor sections. Proliferation and anti-tumor activity of peptide-specific CTL was demonstrated by *in vitro *stimulation with dendritic cells pulsed with the peptides.

**Results:**

Frequency of EGFR-specific CTL correlated significantly with EGFR expression in tumor sections (p = 0.02, r^2 ^= 0.6). Patients with elevated EGFR scores (> 7) had a significantly higher frequency of EGFR-specific CTL than NC and patients with low EGFR scores (< 7). EGFR-specific CTL from cancer patients were expanded *ex vivo *and produced IFN-γ upon recognition of EGFR^+ ^target cells.

**Conclusion:**

EGFR expressed on HNSCC cells induces a specific immune response *in vivo. *Strategies for expansion of EGFR-specific CTL may be important for future immunotherapy of HNSCC patients.

## Background

The transmembrane EGFR protein (HER1/erbB-1) is a member of the erbB family, which also includes the receptor tyrosine kinases HER2 (erbB-2/neu), HER3 (erB-3) and HER4 (erbB-4). Activation of EGFR induces activation of intracellular STAT, MAPK, PI3K and PLC pathways, leading to tumor cell proliferation, angiogenesis, cell migration and a decreased rate of apoptosis [1]. In HNSCC, either over-expression or mutation of EGFR is found in 80-100% of the patients, and both are associated with poor prognosis and decreased survival [2,3]. Therefore, it has been expected that the treatment with EGFR inhibitors, including anti-EGFR antibodies, would be highly successful in inducing tumor regression. However, recently published studies demonstrate that only a small subgroup of HNSCC patients respond to molecular anti-EGFR therapy. Thus, Vermorken et al. recruited 103 patients with disease progression under platinum therapy whose response rate to cetuximab only was 13% [4]. Kirby et al. included 47 HNSCC patients in the palliative gefitinib study with an overall response rate of 8% [5]. Additionally, responses to anti-EGFR therapy seem to be independent of EGFR expression on the surface of tumor cells [6-8].

In order to increase the proportion of patients who benefit from anti-EGFR therapy, new approaches in the field are needed, and due to the central role of EGFR in cancer progression, *ex vivo *expansion and re-injection of autologous EGFR-specific CTL may be one possible potentially attractive alternative. However, in view of the fact that EGFR is a commonly present self-antigen, the existence of circulating EGFR-specific cytotoxic T cells (CTL) may not be taken for granted. The current study aimed to determine the frequency of EGFR-specific CTL in HNSCC patients and to evaluate their specific function *in vitro*.

## Material and methods

### Study design

Peri-operative peripheral blood samples (30 ml) were obtained from 16 HLA-A2.1^+ ^HNSCC patients. Mean age was 62.6 ± 11 years (3 females, 13 males). The control group was age and sex matched (5 females, 11 males) with a mean age of 59.6 ± 9 years. History of cancer in the past was an exclusion factor in the control group. All patients signed a consent form approved by the local ethics committee. Fresh peripheral blood mononuclear cells (PBMC) were isolated by Leucosep^®^-Systems (Greiner, Germany), and stained with monoclonal anti- HLA-A2 antibody BB7.2 - FITC (ATCC, VA) to determine the HLA-A2^+ ^status.

### Peptide-MHC class I complexes

Two EGFR-specific peptides were chosen based on their relevance for cancer progression as previously published [9]. These peptides were used to identify EGFR-specific CTL in the circulation of HNSCC patients and in the control group. The YLN-peptide (YLNTVQPTCV) was used in tetramer form. The KLF-peptide (KLFGTSGQKT) was not available in tetramer form and was therefore used as a pentamer complex (ProImmune, GB). The peptide GILGFVFTL, a dominant peptide of the influenza virus matrix, served as a positive control to identify HLA-A2.1^+ ^individuals, and the peptide ILKEPVHGV, an HIV-1 reverse transcriptase peptide, was used as a negative control. Both peptides were used as tetramers. In order to reduce background staining, control tetramers were titered and used at the lowest possible concentration which still gave a distinctive positive staining in a donor vaccinated for influenza or in an HIV-positive individual [10]. All tetramers were obtained from Beckman Coulter (Germany). For HLA-A2-stabilization assays, peptides were obtained from the Peptide Synthesis Facility, University of Pittsburgh, PA.

### HLA-A2-stabilization assay

In order to determine the binding capacity of the peptides to the HLA-A2 surface complex, T2 cells (500,000/well), which are deficient in 'transporter associated with antigen processing' (TAP 1/2), were co-incubated with various concentrations of the peptides (10 μg, 1 μg, 100 ng/mL) for 18 h in AIM-V medium (Gibco, CA). Cells were then washed with PBS and stained for surface HLA-A2 antigen using anti-HLA-A2 (BB7.2) and goat-anti-mouse secondary antibody. After fixation with paraformaldehyde (2%) cells were then analyzed by single color flow cytometry. For determination of the HLA-A2 binding capacity, the mean fluorescence intensity (MFI) of 'T2 cells + peptide' was divided by the MFI of 'T2 cells alone', resulting in a value between 1 and 2.

### Flow cytometry analysis

PBMC were re-suspended in AIM-V medium, supplemented with Hank's buffered salt solution and fetal calf serum (FCS), and transferred into V-bottom 96-well-plates (Costar, NY) at a concentration of 5 × 10^6 ^cells/well. Tetramers were added (6 μL, diluted 1:40) and washed twice after incubation for 30 min at RT. Aliquots of CD3-FITC, CD8-APC and CD14-PerCP (all Becton Dickinson, Germany) were added and re-suspended in para-formaldehyde (2%) after incubation for 30 min at 4°C.

Approximately 1 × 10^6 ^events, including at least 50,000 gated CD8^+ ^T cells, were acquired using a four-color FACS Calibur Cytometer (Beckton Dickinson). Beckman-Coulter System II software was used for determination of EGFR-specific CTL frequency (CD3^+^CD8^+^CD14^neg^Tetramer^+^). In order to set the gate for tetramer^+ ^events, PBMC were stained with antibodies (CD3/CD8/CD14), but without tetramer. Thus, the gate was set above the mean fluorescence intensity of 28. Every patient and healthy control of this study was stained for HIV tetramer (n = 32). Despite the described gating strategy for CD8^+ ^T cells, we found a low frequency of HIV-tetramer^+ ^events. These events were non-specific by definition, because subjects were presumed HIV negative. The 99^th ^percentile of these HIV-tetramer^+ ^frequencies was calculated using SPSS software (IBM), and it was further used as the lower limit of detection (LLD) of the assay at 0.02%. EGFR-specific tetramer frequencies below this LLD were considered negative. These findings were in agreement with our previous experiences [11,12], and in this study, all HNSCC patients with an EGFR score > 7 had EGFR-specific tetramer frequencies well above the LLD.

### Immunohistochemistry (IHC)

Paraffin blocks of tumor samples were provided by the Department of Pathology, Duesseldorf, Germany, and the diagnosis of HNSCC was confirmed in each case by a pathologist (R.E.). Representative tumor sections containing areas of invasive HNSCC were selected for IHC. Normal tissues at the edges of the tumor samples served as an internal non-tumor control. For IHC, formalin-fixed, paraffin-embedded tumor tissues were sectioned at 5 μm. Sections were air-dried overnight at 37°C, deparafinized and dehydrated. The EGFR-positive cell line UD-SCC-8 served as a positive control. After antigen retrieval and inactivation of endogenous peroxidase, the sections were stained with a mAb against EGFR (Clone 8C9, Zymed Lab, Germany) and Vectastain-Elite-ABC kit (Vector Laboratories, CA). Counterstaining was provided by Mayer's hemalum.

Staining intensity was evaluated on paraffin-embedded tumor sections by microscopy using a scale from 1 to 4: 1 = very low, 2 = low, 3 = medium, and 4 = high staining intensity. The frequency of EGFR-positive cells was scored as follows: 0 = no positive cells, 1 = less than 10%, 2 = 10 - 50%, 3 = 51 - 80% and 4 = 81 - 100% EGFR-positive tumor cells. The EGFR score (0-16) was calculated as the product of staining intensity multiplied by the number of EGFR-positive cells. The lowest score obtained in the examined sections was 1 with <10% of cells showing a very low staining intensity. The highest score was 12 with ≤ 80% of cells showing a high staining intensity or with >80% of cells showing a medium staining intensity. In order to rule out subjective influences in the evaluation of the EGFR-score, evaluation was performed in a blinded setting. The pathologist was not aware of the frequency of EGFR-peptide specific CTL when evaluating the tumor samples.

### In vitro expansion of anti-EGFR-specific T cells

Human dendritic cells (DC) were generated according to a modified method of Sallustro and Lanzavecchia [13]. Briefly, PBMC of HNSCC patients were incubated for 2 h at 37°C in AIM-V medium, and non-adherent cells were removed by gentle washing with warm medium. The remaining plastic adherent cells were incubated in AIM-V medium (Gibco, CA) with 1,000 U/ml granulocyte macrophage colony stimulating factor (GM-CSF, Immunex, WA) and 1,000 U/ml IL-4 (Schering, NJ). Immature DC were harvested on day 6 with cold Hank's solution and 6 ml Trypsine (Gibco, CA) and used as antigen presenting cells (APC). DC were re-suspended at the concentration of 2 × 10^6 ^cells/ml in PBS containing 10 μg/ml of peptide and incubated at 37°C for 45 min. Subsequently, 0.3 × 10^6 peptide-pulsed DC were co-cultured with 1 × 10^6 PBMC in 24-well tissue culture plates (Costar, NY) in a final volume of 2 ml/well of X-Vivo medium (Cambrex, Germany). IL-7 (25 ng/ml, BD Biosciences) was added for the first 72 h and, additionally, IL-2 (20 IU/ml, Chiron, Germany) was added for the remaining time in culture. The lymphocytes were re-stimulated weekly with 0.3 × 10^6 peptide-pulsed autologous DC and harvested after the third cycle (day 21).

### Culture of target cell lines

Target cells included HLA-A2.1^+ ^EGFR-positive laryngeal carcinoma cell line UD-SCC-8 and HLA-A2.1^+ ^laryngeal carcinoma cell line HLac79 with low expression of EGFR, kindly provided by Prof. Bier and Prof Zenner, respectively [14]. Previous ELISA-experiments have shown an EGFR expression, which was 675-fold higher in the cell line UD-SCC-8 (13,498 fmol/mg protein) compared to the cell line HLac79 (20 fmol/mg protein, Calbiochem Merck, Germany). Cells were grown in plastic culture flasks (Greiner, Germany) under standard conditions (37°C, 5% CO2, 100% humified), using modified Eagle's medium supplemented with 10% heat-inactivated FBS, 2 mM L-glutamine, 50 μg/ml streptomycin and 50 IU/ml penicillin (all ICN, Germany), as described previously. To transfer or passage the cell lines, almost confluent monolayers were detached with 0.05% trypsin and 0.02% EDTA solution (Boehringer, Germany). Subsequently, cells were washed twice in medium and re-suspended in culture flasks.

### Enzyme-linked ELISPOT assay for IFN-γ

Reactivity of the generated effector cells against the EGFR-peptides YLN and KLF was tested by the IFN-γ ELISPOT assay. Patients' PBMC were stimulated with autologous, peptide-loaded DC in order to test their ability to respond to the cognate epitope *in vitro*. The ELISPOT assay was performed in 96-well plates (Nunc, Denmark). The capture and detection Abs and AEC substrate reagent were purchased from BD Biosciences (Human IFN-γ ELISPOT Pair, AEC Substrate Reagent Set). For antibody blocking experiments, target cells were pre-incubated for 30 min with 10 μg/ml anti-HLA class I-specific monoclonal Ab, W6/32 (HB95, ATCC) or respective IgG2 isotype control (BD Pharmingen, CA). Additionally, the experiments were repeated with target cell lines which were loaded with EGFR-peptides. Spots were counted by two independent investigators (K.S., P.B.). The ratio of effector and target cells was 1:1 with 10,000 cells/well for each group. The specificity of generated CTL was confirmed by tetramer staining.

### Statistical analysis

Tetramer-positive cells were quantified by flow cytometry and expressed as percent of CD8^+ ^T cells. Averages were calculated as geometric means. For non-parametric distribution of samples, p-values were calculated by Kruskal-Wallis and two-tailed exact Wilcoxon-Mann-Whitney tests using SPSS software (IBM). Deviations were presented as standard error of the mean. Correlations were calculated by Spearman tests. P values < 0.05 and r^2 ^values > 0.5 were considered to be significant.

## Results

### HLA-A2 binding assay

**Figure 1:**
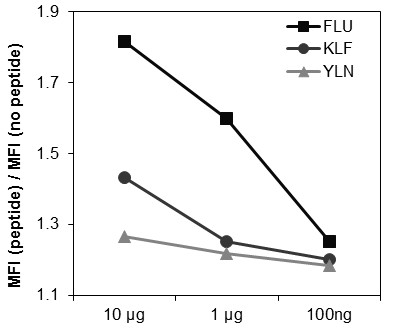
**Binding capacity of EGFR-specific peptides to HLA-A2 complex on the surface of T2 cells**. The FLU peptide showed the best binding capacity to surface HLA-A2 compared to the two EGFR-specific peptides KLF and YLN. In all peptides expression of surface HLA-A2 complex was dose-dependent.

The ability of the EGFR-specific peptides (KLF, YLN) to stabilize the HLA-A2 complex on the surface of TAP-deficient T2 cells in relation to the FLU peptide is shown in Figure 1. In the T2-assay, stabilisation of the HLA-A2 complex correlates well with the binding capacity of the specific peptide to HLA-A2, which validates the peptide for recognition of EGFR-specific CTL. In this assay, the KLF peptide had a higher binding capacity (79% of FLU) than the YLN peptide (70% of FLU). For all three peptides, expression of surface HLA-A2 decreased in correlation to the peptide concentration. Additionally, the sequences of both EGFR-peptides, as well as the control peptides (FLU and HIV) were entered into the web-based program for peptide binding prediction from NIH (http://www-bimas.cit.nih.gov/molbio/). The scores for the EGFR-peptides YLN (320) and KLF (96) were higher than for HIV (39) but lower than for FLU (550). The predicted binding ability was in accordance to our present results, in which the frequency of EGFR-specific T cells was slightly higher for the YLN peptide compared to the KLF-peptide. For comparison, we also entered a peptide from the HPV1a-protein (ILSRFKDTA) into the binding prediction program, which had been described to have a low affinity to HLA-A2 [15]. The score for the HPV1a-protein was 15.

### Frequency of EGFR-specific CD8^+ ^T cells

**Figure 2:**
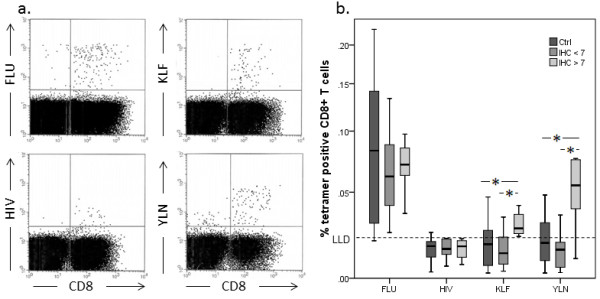
**Mean frequencies of CD8^+ ^EGFR-specific T cells in HNSCC patients and healthy controls**. Representative dot plots for tetramer staining are displayed **(a)**. Results are displayed in percent (%) for the control staining with FLU- and HIV-specific tetramers, as well as the EGFR-specific pentamer KLF and tetramer YLN. For both EGFR-specific peptides, HNSCC patients with a high EGFR score (> 7) had a significantly elevated frequency of EGFR-specific CTL, compared to patients with low EGFR score (< 7) and normal donors (* p-value < 0.05). No significant difference was seen between normal donors and patients with low EGFR score **(b)**.

In HNSCC patients with high EGFR score (> 7), the frequency of EGFR-specific CD8^+ ^T cells was significantly (p < 0.05) increased for both peptides compared to NC and HNSCC patients with a low EGFR score (< 7) (Figure 2). Also, the frequencies of CTL for both peptides were correlated significantly in all individuals (r^2 ^= 0.5). Patients' characteristics and frequencies of CTL are shown in Table 1. No correlation was found between EGFR-specific CTL frequency and TNM status or gender of the patients.

**Table 1:** Patients' age, gender, tumor staging, frequencies of EGFR-specific T cells ^a^ and EGFR score ^b^

No	Age (yrs)	TNM	EGFR score	KLF (%)	YLN (%)
1	76	T4 N0 M0	1	0.0029	0.0027
2	84	T3 N1 MX	2	0.0125	0.0084
3	59	T4 N0 MX	2	0.0034	0.0074
4	61	T1 N0 MX	4	0.0050	0.0143
5	55	T2 N0 MX	4	0.0145	0.0193
6	56	T3 N2b M0	4	0.0216	0.0164
7	83	T2 N0 MX	4	0.0107	0.0241
8	67	T1 N0 M0	4	0.0069	0.0061
9	41	T2 N2a MX	4	0.0089	0.0045
10	68	T3 N2 M0	6	0.0062	0.0045
11	55	T2 N2 MX	6	0.0134	0.0145
	**64.1**		**3.7**	**0.015 ± 0.001**	**0.014 ± 0.002**
12	58	T3 N1 M0	9	0.0265	0.0758
13	52	T4 N2b M0	9	0.0351	0.0387
14	64	T3 N1 M0	12	0.0218	0.0097
15	60	T3 N0 MX	12	0.0409	0.0549
16	62	T2 N2a MX	12	0.0234	0.0748
	**59.2**		**10.8**	**0.029 ± 0.008**	**0.051 ± 0.028**

a - The frequency of EGFR-specific T cells in PBMC are shown for KLF (pentamer) and YLN (tetramer) peptides in percent (%) of CD8 positive T cells in HNSCC patients with low EGFR score (2-6) and high EGFR score (9-12). All mean values are printed in bold for each patient group.

b - EGFR scores were calculated as staining intensity (1-4) multiplied by the number of EGFR-positive cells (0-4) determined by immunohistochemistry.

### Immunohistochemistry

**Figure 3:**
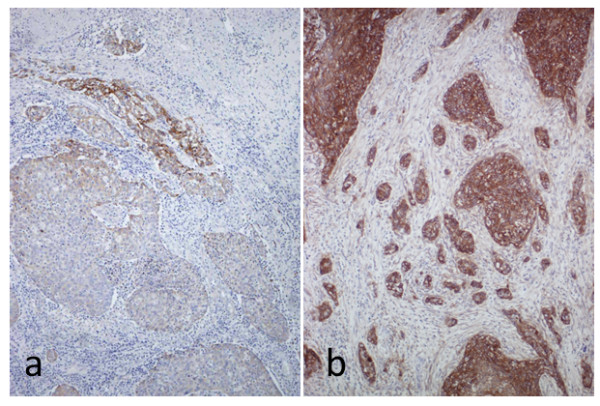
**Staining for EGFR in representative HNSCC samples**. The EGFR score (0-16) in tumor samples was calculated as a product of staining intensity (1-4) multiplied by the percentage of positive cells (0-4). Tumor sample with low EGFR expression and EGFR score 2 **(a)**. Tumor sample with high EGFR expression and EGFR score 12 **(b)**. Homogenous expression of EGFR was found in the membranes and cytoplasm of tumor cells (Mag. × 100).

**Figure 4:**
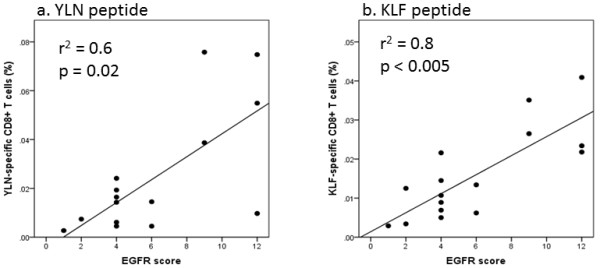
**Correlations between the EGFR score and frequency of EGFR-specific CD8^+ ^T cells in PBMC**. The frequency of EGFR-specific T cells in PBMC of HNSCC patients is given in percent (%) of CD8^+ ^T cells for YLN-tetramer **(a) **and KLF-pentamer **(b)**. The EGFR score reflected the staining intensity and frequency of EGFR positive cells in corresponding tumor samples.

All tumor samples were positive for EGFR, and 5 samples showed an EGFR score of 9 or higher. A homogenous ABC-dye uptake was found in tumor cell membranes and cytoplasm of all tumor samples as seen in Figure 3. This staining pattern conformed to staining patterns obtained in the EGFR-positive cell line, UD-SCC-8, which served as a positive control. In the negative control tissues, EGFR expression was observed only in the basal epithelial layers (not shown). Comparing the EGFR scores with the frequency of EGFR-specific CTL revealed a strong positive correlation for both the YLN-peptide (p = 0.02, r^2 ^= 0.6) and the KLF-peptide (p < 0.005, r^2 ^= 0.8) as seen in Figure 4. A clear cut-off was located between the EGFR scores of 6 and 9. None of the early stage tumors (T1) displayed an EGFR-score above 4. For the other tumors (T2-4) samples could be subdivided into weak or strong EGFR expression.

### CTL ability to recognize EGFR^+ ^target cells

**Figure 5:**
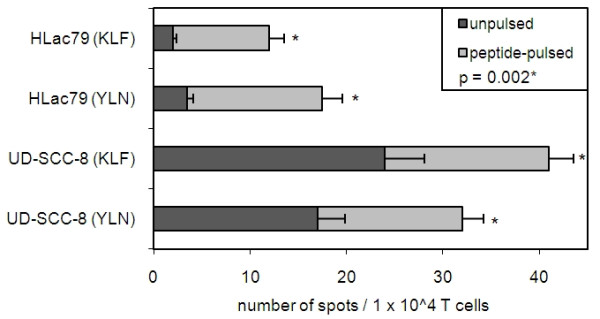
**EGFR-specific CTL recognize tumor cells**. CTL were generated from PBMC of HNSCC patients in co-cultures with autologous DC loaded with EGFR-peptides KLF or YLN. T cells were tested against the EGFR-positive cell line UD-SCC-8 and the EGFR-negative cell line HLac79, which were either unpulsed or pulsed with the EGFR peptides. The T cell: target cell ratio was 1:1. The data show the mean number of counted spots for unpulsed and pulsed cell lines obtained using CTL generated from PBMC of HNSCC patients. Pulsing cell lines with peptide significantly increased IFN-γ production in CTL (* p = 0.002). Background in the unpulsed EGFR-negative cell line HLac79 was not subtracted.

After *in vitro *expansion, PBMC of HLA-A2.1^+ ^patients were tested for reactivity against the EGFR-positive cell line (UD-SCC-8) and the EGFR-negative cell line (HLac79) in INF-γ ELISPOT experiments (n = 3). Both target cell lines were used unpulsed or after pulsing with EGFR-peptides. INF-γ-secretion was significantly increased by pulsing target cells with EGFR-peptides (p = 0.002). Consequently, for both peptides, the highest IFN-γ-secretion was observed in the EGFR-positive cell line UD-SCC-8 which was additionally pulsed with EGFR-peptide (32 ± 3 spots for YLN, 41 ± 3 spots for KLF/1 × 10^5 cells). Pulsing target cells with the KLF-peptide increased INF-γ-production by 14 ± 1 spots/1 × 10^5 cells. The increase was 15 ± 1 spots/1 × 10^5 cells, when cells were pulsed with the YLN-peptide. Specificity of CTL for the EGFR-peptides was confirmed by the observation that IFN-γ-secretion was almost undetectable in the unpulsed EGFR negative cell line HLac79, and only pulsing the target cells with the EGFR peptides increased IFN-γ-secretion by 6-fold. The results of ELISPOT assays are shown in Figure 5. Frequencies of EGFR-peptide specific CTL were compared before and after *in vitro *expansion. For the KLF-peptide, the frequency before expansion was 0.02 - 0.04% of CD8^+ ^T cells in HNSCC patients with high EGFR score (>7). After *in vitro *expansion, the frequency of EGFR-peptide specific CTL was 20 spots/10,000 cells, corresponding to 0.2 ± 0.03% of PBMC. The effect of expansion was similar for YLN-peptide specific CTL.

## Discussion

As EGFR is a self-antigen, the frequency of EGFR-specific CTL is expected to be low in the peripheral blood of HNSCC patients, and the ability of these cells to recognize EGFR^+ ^tumor cells to be weak. Using a sensitive and specific method available for the detection of rare peptide-specific T cells, we have been successful in establishing that EGFR-specific CD8^+ ^T cells are present in the circulation of HNSCC patients with high EGFR scores. Additionally, the frequency of EGFR-specific CTL in the peripheral blood of HNSCC patients correlated strongly with the EGFR expression on tumor samples. This correlation suggests that EGFR over-expression on the tumor cells clearly induces T cell responses in the periphery. Interestingly, only the combination of advanced tumor size (T2-4) and high EGFR score (> 7) was followed by a significant increase of EGFR-specific CTL. In case of small tumors (T1), the total amount of EGFR antigen expressed on the cell surface is probably insufficient to induce immune T cell response. In conclusion, the host immune response is too slow to inhibit tumor growth in its early stage. Moreover, it has been reported that suboptimal antigen doses presented by DC induce the development of Treg, while high antigen doses favor development of effector T cells [16].

Additionally to confirming the existence of EGFR-specific CTL, we have succeeded in expanding autologous EGFR-specific T cells of HNSCC patients *in vitro. *Expanded EGFR-specific CTL recognized EGFR on the surface of target cells, irrespective of whether these targets actively expressed the peptide or if they were exogenously loaded with EGFR peptides. This finding introduces the option for expanding EGFR-specific CTL *ex vivo *for adoptive immunotherapy of HNSCC in addition to conventional surgery and chemo-radiotherapy [17].

The current results complement our earlier studies of cellular immune responses to other tumor-associated antigens, such as wild-type p53 peptides and HPV-16. Using the tetramer-based technique in previous studies, elevated frequencies of HPV-specific CTL were detected in HNSCC patients with HPV/p16^+ ^tumors [18]. In another tetramer-based study, the frequency of CTL specific for the self-antigen p53 showed an inverse correlation to p53 expression in the tumor. The frequency of p53-specific CTL was increased in HNSCC patients whose tumors had a normal p53 expression, whereas it was decreased in tumors with high p53 expression, which was explained by epitope loss under immune pressure [11]. Further tetramer studies indicated that p53-specific CTL decreased in the peripheral blood after surgery of HPV^+ ^HNSCC but not in HPV-negative HNSCC [19]. The detection of EGFR-specific CTL in the circulation of HNSCC is in line with other studies which used different EGFR-specific peptides. Andrade et al. found, that treatment of tumor cells with cetuximab increased their recognition by EGFR-specific CTL *in vitro *[20].

Despite the elevated frequency of EGFR-specific CTL in the circulation of HNSCC patients with high EGFR score, tumor growth was not inhibited. These results are counterintuitive but may be explained by one or more of the following events: **(a) **the EGFR-peptide is presented in association with HLA-A2.1 on the tumor surface in a confirmation unrecognizable by T cells [21], or DC in tumor-bearing individuals might have impaired antigen presenting capability [22]. Consequently, adaptive immune responses to the tumor peptides are inefficient, and frequencies of EGFR-specific CTL remain low. **(b) **Alternatively, apoptosis of tumor-specific T cells might be responsible for their low frequencies. As shown by Albers et al., annexin expression, which indicates apoptosis, is increased in wild-type p53-specific CTL compared to non-tumor specific T cells in HNSCC [12]. **(c) **The presence of tumor-induced suppression in HNSCC patients, as evidenced by increased proportions of myeloid derived suppressor cells, tumor-derived microvesicles, and regulatory T cells at the tumor site and in the peripheral circulation may account for lack of immune responses to EGFR peptides [23]. **(d) **Not only antigen presentation on the cell surface, but also the intracellular turnover of the protein might determine and modulate antigen recognition by the immune system, as observed for p53 [24]. As p53 and EGFR both are self-antigens, this might also be true for EGFR recognition. Nevertheless, despite these various difficulties, EGFR-specific CTL were detectable in the peripheral blood of HNSCC patients and could be expanded *in vitro*. Importantly, we found a strong correlation of specific T cell frequency and EGFR expression on tumor cells. Thus, the impairment most likely accounting for the insufficient EGFR-specific immune response in HNSCC patients might be related to the dose of antigen and tumor-derived immune suppression. Considering the presented results, the number of EGFR-specific CTL before and after tumor therapy in correlation to the frequency of regulatory T cells would be of high interest and will be addressed in future longitudinal studies. Further, our results suggest that subsequent studies of tumor therapy should not be limited to the monitoring of tumor regression. They should also focus on the effect which therapy has on various cell populations of the immune system, including regulatory T cells, MDSC, Th-17-cells, and antigen-specific CTL as well as their cytokine expression profile.

## Conclusions

EGFR expressed on HNSCC cells induces a specific immune response *in vivo. *Strategies for expansion of EGFR-specific CTL may be important for future immunotherapy of HNSCC patients.

## Abbreviations

CTL: cytotoxic T cell; DC: dendritic cell; EGFR: epidermal growth factor receptor; HIV: human immunodeficiency virus; HNSCC: head and neck squamous cell cancer; IVS: in vitro stimulation; MAPK: mitogen-activated protein kinase; MFI: mean fluorescence intensity; NC: normal control; PBMC: peripheral blood mononuclear cells; PI3K: phosphatidylinositol 3-kinase; PLC: phospholipase C; STAT: signal transducers and activators of transcription;

## Authors' contributions

PJS Wrote the manuscript, data analysis, PB ELISPOT assays, RE pathological analysis of tumor slides, EB sample collection and patient selection, MB Tetramer binding assay, JG sample collection and patient selection, CAD performed experiments, GFL sample collection and patient selection, RLF edited the manuscript, PAA performed experiments, SB edited the manuscript, SL provided clinical management and edited the manuscript, TLW provided valuable discussion and wrote manuscript, TKH provided study design, wrote manuscript. All authors have read and approved the final manuscript.

## Competing interests

The authors declare that they have no competing interests.
